# Study of multi-dimensional problems arising in wave propagation using a hybrid scheme

**DOI:** 10.1038/s41598-024-56477-5

**Published:** 2024-03-10

**Authors:** Jinxing Liu, Muhammad Nadeem, M. S. Osman, Yahya Alsayaad

**Affiliations:** 1 Faculty of Science, Yibin University, Yibin, 644000 China; 2 School of Mathematics and Statistics, Qujing Normal University, Qujing, 655011 China; 3 Department of Mathematics, Faculty of Science, Cairo University, Giza, 12613 Egypt; 4 Department of Physics, Hodeidah University, Al-Hudaydah, Yemen

**Keywords:** Sawi integral transform, Homotopy perturbation scheme, Multi-dimensional wave equations, Approximate solutions, Applied mathematics, Fluid dynamics

## Abstract

Many scientific phenomena are linked to wave problems. This paper presents an effective and suitable technique for generating approximation solutions to multi-dimensional problems associated with wave propagation. We adopt a new iterative strategy to reduce the numerical work with minimum time efficiency compared to existing techniques such as the variational iteration method (VIM) and homotopy analysis method (HAM) have some limitations and constraints within the development of recurrence relation. To overcome this drawback, we present a Sawi integral transform ($\mathbb {S}$T) for constructing a suitable recurrence relation. This recurrence relation is solved to determine the coefficients of the homotopy perturbation strategy (HPS) that leads to the convergence series of the precise solution. This strategy derives the results in algebraic form that are independent of any discretization. To demonstrate the performance of this scheme, several mathematical frameworks and visual depictions are shown.

## Introduction

Several notable advances in computational approaches have been developed for engineering and scientific applications, including geometrical description, flexible artificial materials, and acoustic wave propagation^1–3^. Partial differential equations (PDEs) have a significant impact on many scientific and engineering fields, including electronics, hydrodynamics, computational motion, physical biology, the engineering of chemicals, dietary fiber, mechanics, material dynamics, and geometrical optics^4–7^. Numerous researchers have investigated different methods to derive the analytical results for such PDEs. Utilizing a meshfree approach named the Radial basis function pseudo spectral (RBF-PS) method, researchers numerically examined the solutions for both integer and fractional KdV type equations on a finite domain with periodic boundary conditions^8,9^. Although the computations associated with these approaches are fairly straightforward and certain variables are based on the assumption of a variety of limitations. As a result, many scientists are looking for new techniques to overcome these restrictions. Numerous scientists and other researchers have offered several methods for assessing the analytical findings^10–12^. Several academics and scientists have used HPS^13,14^ to solve complicated physical problems. When employing this method, the solution series converges relatively quickly in most cases. The authors^15,16^ used HPS to the oscillation challenges in nonlinearity and demonstrated its effectiveness in providing analytical findings.

The wave problem is a partial differential equation for a scalar function offering wave propagation in the motion of fluids. Wazwaz^17^ used the VIM to study linear and nonlinear problems. Ghasemi et al.^18^ computed the effective results for two-dimensional nonlinear differential problem using HPS. Keskin and Oturanc^19^ proposed a new method for the analytical results of wave problems. Dehghan et al.^20^ applied HAM to derive the approximation results for PDEs. Ullah et al.^21^ proposed a homotopy optimum technique to generate algebraic findings for wave challenges. Thorwe and Bhalekar^22^ used Laplace transform method to obtain approximation solution of partial integro-differential equations. Adwan et al.^23^ presented analytical findings for multidimensional wave challenges and validated the proposed technique. The HPS was applied for the approximate solutions of wave equations by Jleli et al.^24^. The researchers in^25^ proposed the finite element technique and separated the wave system to derive their approximate solution. These approaches include a lot of limitations and assumptions during the estimation of problems.

The current study aims to use a new iterative technique for multi-dimension challenges by combining $\mathbb {S}$T and HPS. In the present work, we eliminate these drawbacks and constraints by offering a novel iterative method for these multi-dimensional wave issues. An iteration series with approximate findings that are close to the precise outcomes is produced by this new strategy. This technique performs more effectively and produces more appealing outcomes for the present challenges. The following is a description of this work: the concept of Sawi integral transform is given in “Fundamental concepts”. In “Formulation of new iterative strategy”, we build our new strategy to achieve the multi-dimension model findings. The convergence theorem has been laid out in “Convergence of new iterative strategy”. In “Numerical applications”, a few numerical examples are examined to demonstrate the power of new technique and we offer the conclusion at the end of “Conclusion remarks and future work”.

## Fundamental concepts

In this portion, we give few fundamental features of $\mathbb {S}$T that are helpful in the development of our new strategy.

### Sawi transform

#### Definition 2.1

Let $\vartheta$ be a function of $\eta \ge 0$. Then, $\mathbb {S}$T is^26,27^

1 $$\begin{aligned} \mathbb {S}[\vartheta (\eta )]=Q(\theta )=\frac{1}{\theta ^{2}}\int _{0}^{\infty }\vartheta (\eta ) e^{-\dfrac{\eta }{\theta }} dt.\ \ \ \eta \ge 0,\ \ \ k_{1}\le \theta \le k_{2} \end{aligned}$$

in which $\mathbb {S}$ represents the symbol of $\mathbb {S}$T. Now

$$\begin{aligned} \mathbb {S}^{-1}[Q(\theta )]=\vartheta (\eta ), \ \ \ \ \ \mathbb {S}^{-1} \ \text {is the inverse }\mathbb {S}\text {T,} \end{aligned}$$

where $Q(\theta )$ shows the function of $\vartheta (\eta )$. The $\mathbb {S}$T of $\vartheta (\eta )$ for $\eta \ge 0$ exist if $\vartheta (\eta )$ tends to exponentially ordered and piecewise continuous. The existence of $\mathbb {S}$T for $\vartheta (\eta )$ is basically predicated on the two requirements mentioned.

#### Proposition 1

*Now, we define the basic propositions of* $\mathbb {S}$T. *Therefore, let* $\mathbb {S}\{\vartheta _{1}(\eta )\}=Q_{1}(\theta )$ *and* $\mathbb {S}\{\vartheta _{2}(\eta )\}=Q_{2}(\theta )$^28,29^, thus

2 $$\begin{aligned} \begin{aligned} \mathbb {S}\{a \vartheta _{1}(\eta )+b \vartheta _{2}(\eta )\}&=a \mathbb {S} \{\vartheta _{1}(\eta )\}+b \mathbb {S} \{\vartheta _{2}(\eta )\},\\\Rightarrow \ \ \ \mathbb {S}\{a \vartheta _{1}(\eta )+b \vartheta _{2}(\eta )\}&=a Q_{1}(\theta )+b Q_{2}(\theta ), \end{aligned} \end{aligned}$$

#### Proposition 2

*Now, for the differential characteristics of* $\mathbb {S}$T, *we consider* $\mathbb {S}\{\vartheta (\eta )\}=Q(\theta )$, *the differential characteristics are defined as*^30^

3 $$\begin{aligned} \begin{aligned} \mathbb {S}\{\vartheta '(\eta )\}&=\dfrac{Q(\theta )}{\theta }- \dfrac{\vartheta (0)}{\theta ^{2}},\\ \mathbb {S}\{\vartheta ''(\eta )\}&=\dfrac{Q(\theta )}{\theta ^{2}}- \dfrac{\vartheta (0)}{\theta ^{3}}-\dfrac{\vartheta '(0)}{\theta ^{2}},\\ \mathbb {S}\{\vartheta ^{m}(\eta )\}&=\dfrac{Q(\theta )}{\theta ^{m}}- \dfrac{\vartheta (0)}{\theta ^{m+1}}- \dfrac{\vartheta '(0)}{\theta ^{m}}-\cdots - \dfrac{\vartheta ^{m-1}(0)}{\theta ^{2}}. \end{aligned} \end{aligned}$$

## Formulation of new iterative strategy

This section examines the approximate solutions of 1D, 2D, and 3D wave problems by using new iterative strategy (NIS). This approach can be used to solve differential equations based on initial conditions. We stated that the construction of this approach does not depend on integrating and other suppositions. Let a differential equation like that

4 $$\begin{aligned} \vartheta ''(x_{1},\eta )+\vartheta (x_{1},\eta )+f(\vartheta )=f(x_{1},\eta ), \end{aligned}$$

subjected to initial conditions

5 $$\begin{aligned} \vartheta (x_{1},0)=a_{1},\qquad \vartheta _{\eta }(x_{1},0)=a_{2} \end{aligned}$$

where $f(\vartheta )$ denotes the nonlinear element, $f(x_{1},\eta )$ is known component of arbitrary constants $a_{1}$ and $a_{2}$, and $\vartheta (x_{1},\eta )$ is a uniform function. Moreover, we may express Eq. (4) like this:

6 $$\begin{aligned} \vartheta ''(x_{1},\eta )=-\vartheta (x_{1},\eta )-f(\vartheta )+f(x_{1},\eta ). \end{aligned}$$

A function of a real variable can be transformed into an expression of a complex variable using an integral transformation known as the Sawi transform in mathematics. This transformation has several uses in the fields of science and technology because it serves as a tool to deal with differential problems.

Apply $\mathbb {S}$T on Eq. (6), we get

$$\begin{aligned} \mathbb {S}[\vartheta ''(x_{1},\eta )]=\mathbb {S}[-\vartheta (x_{1},\eta )-g(\vartheta )+g(x_{1},\eta )]. \end{aligned}$$

Using the formula as defined in Eq. (3), it yields

$$\begin{aligned} \frac{Q(\theta )}{\theta ^{2}}-\frac{\vartheta (0)}{\theta ^{3}}-\frac{\vartheta '(0)}{\theta ^{2}}=-\mathbb {S}[\vartheta (x_{1},\eta )+f(\vartheta )-f(x_{1},\eta )]. \end{aligned}$$

Thus, $Q(\theta )$ is derived as

7 $$\begin{aligned} Q[\theta ]=\frac{\vartheta (0)}{\theta }+\vartheta '(0)-\theta ^{2} \mathbb {S}[\vartheta (x_{1},\eta )+f(\vartheta )-f(x_{1},\eta )]. \end{aligned}$$

On inverse $\mathbb {S}$T on Eq. (7), we get

$$\begin{aligned} \vartheta (x_{1},\eta )&=\vartheta (0)+\eta \vartheta '(0)-\mathbb {S}^{-1}\Big [\theta ^{2} \mathbb {S}\Big \{\vartheta (x_{1},\eta )+f(\vartheta )-f(x_{1},\eta )\Big \}\Big ]. \end{aligned}$$

Use the condition (5), we obtain

8 $$\begin{aligned} \vartheta (x_{1},\eta )&=\vartheta (x_{1},0)+ \eta \vartheta _{\eta }(x_{1},0)+\mathbb {S}^{-1}\Big [\theta ^{2} \mathbb {S}\Big (f(x_{1},\eta )\Big )\Big ]-\mathbb {S}^{-1}\Big [\theta ^{2} \mathbb {S}\Big (\vartheta (x_{1},\eta )+f(\vartheta )\Big )\Big ], \end{aligned}$$

This Eq. (8) is known as the development of NIS of Eq. (4).

Let HPS be introduced as

9 $$\begin{aligned} \vartheta (\eta )=\sum _{i=0}^{\infty }p^{i}\vartheta _{i}(n) =\vartheta _{0}+p^{1}\vartheta _{1}+p^{2}\vartheta _{2}+\cdots , \end{aligned}$$

where as the nonlinear variable $f(\vartheta )$ is stated as

10 $$\begin{aligned} f(\vartheta )=\sum _{i=0}^{\infty }p^{i}H_{i}(\vartheta ) =H_{0}+p^{1}H_{1}+p^{2}H_{2}+\cdots . \end{aligned}$$

Hence, we are able to generate $H_{n}'s$ polynomial as

11 $$\begin{aligned} H_{n}(\vartheta _{0}+\vartheta _{1}+\cdots +\vartheta _{n}) =\frac{1}{n!}\frac{\partial ^{n}}{\partial p^{n}}\left( f\left( \sum _{i=0}^ {\infty } p^{i}\vartheta _{i}\right) \right) _{p=0}, \ \ \ \ n=0,1,2,\cdots \end{aligned}$$

Use Eqs. (9)–(11) in Eq. (8) and evaluate the similar components of *p*, it yields

$$\begin{aligned} p^{0}&:\vartheta _{0}(x_{1},\eta )=G(x_{1},\eta ),\\ p^{1}&:\vartheta _{1}(x_{1},\eta )=-\mathbb {S}^{-1}\Bigg [\theta ^{2} \mathbb {S}\bigg \{\vartheta _{0}(x_{1},\eta )+H_{0}(\vartheta )\bigg \}\Bigg ],\\ p^{2}&:\vartheta _{2}(x_{1},\eta )=-\mathbb {S}^{-1}\Bigg [\theta ^{2} \mathbb {S}\bigg \{\vartheta _{1}(x_{1},\eta )+H_{1}(\vartheta )\bigg \}\Bigg ],\\ p^{3}&:\vartheta _{3}(x_{1},\eta )=-\mathbb {S}^{-1}\Bigg [\theta ^{2} \mathbb {S}\bigg \{\vartheta _{2}(x_{1},\eta )+H_{2}(\vartheta )\bigg \}\Bigg ],\\&\vdots . \end{aligned}$$

Following this procedure, which results in

12 $$\begin{aligned} \vartheta (x_{1},\eta )=\vartheta _{0}+\vartheta _{1} +\vartheta _{2}+\cdots =\sum _{i=0}^{\infty }\vartheta _{i}. \end{aligned}$$

Hence, Eq. (12) provides a closed-form approximation to the differential problem.

## Convergence of new iterative strategy

### Theorem 4.1

*Let* $[a,b]\times [0,T]$ *be the rectangular interval on which the Banach space* $B\equiv C([a,b]\times [0,T])$ *is defined. Then Eq*. (12) $\vartheta (x_{1},\eta )=\sum _{i=0}^{\infty }\vartheta _{i}(x_{1},\eta )$ *is convergent series, if* $\vartheta _{0}\in B$ *is bounded and* $\left\| \vartheta _{i+1}\right\| \le \left\| \vartheta _{i}\right\| , \forall \vartheta _{i} \in B$, *and for* $0<\delta <1$.

### Proof

Taking the series $\left\{ F_r\right\}$ as a partial sum of Eq. (12), we obtain

13 $$\begin{aligned} \begin{aligned} F_0&=\vartheta _0(x_{1}, \eta ), \\ F_1&=\vartheta _0(x_{1}, \eta )+\vartheta _1(x_{1}, \eta ), \\ F_2&=\vartheta _0(x_{1}, \eta )+\vartheta _1(x_{1}, \eta )+\vartheta _2(x_{1}, \eta ), \\&\vdots \\ F_r&=\vartheta _0(x_{1}, \eta )+\vartheta _1(x_{1}, \eta )+\vartheta _2(x_{1}, \eta )+\ldots +\vartheta _r(x_{1}, \eta ) . \end{aligned} \end{aligned}$$

Next, we establish that $\left\{ F_r\right\} _{r=0}^{\infty }$ is a Cauchy sequence in *B* in order to validate this theorem. Therefore,

14 $$\begin{aligned} \begin{aligned} \left\| F_{r+1}-F_r\right\|&=\left\| \vartheta _{r+1}(x_{1}, \eta )\right\| , \\&\le \delta \left\| \vartheta _r(x_{1}, \eta )\right\| , \\&\le \delta ^2\left\| \vartheta _{r-1}(x_{1}, \eta )\right\| , \\&\vdots \\&\le \delta ^{r+1}\left\| \vartheta _0(x_{1}, \eta )\right\| . \end{aligned} \end{aligned}$$

Hence, for any pair $r, n \in N$, where $r>n$, we have

15 $$\begin{aligned} \begin{aligned} \left\| F_r-F_n\right\|&=\left\| \left( F_r-F_{r-1}\right) +\left( F_{r-1}-F_{r-2}\right) +\left( F_{r-2}-F_{r-3}\right) +\ldots +\left( F_{n+1}-F_n\right) \right\| , \\&\le \left\| F_r-F_{r-1}\right\| +\left\| F_{r-1}-F_{r-2}\right\| +\left\| F_{r-2}-F_{r-3}\right\| +\ldots +\left\| F_{n+1}-F_n\right\| , \\&\le \delta ^r\left\| \vartheta _0(x_{1}, \eta )\right\| +\delta ^{r-1}\left\| \vartheta _0(x_{1}, \eta )\right\| +\ldots +\delta ^{n+1}\left\| \vartheta _0(x_{1}, \eta )\right\| , \\&\le \beta \left\| \vartheta _0(x_{1}, \eta )\right\| . \end{aligned} \end{aligned}$$

where $\beta =\frac{\left( 1-\delta ^{r-n}\right) }{(1-\delta )} \delta ^{n+1}$. Since $\vartheta _0(x_{1}, \eta )$ is bounded, therefore $\left\| \vartheta _0(x_{1}, \eta )\right\| <\infty$. As *n* grows and $n \rightarrow \infty$ leads to $\beta \rightarrow 0$ for $0<\delta <1$, so

16 $$\begin{aligned} \lim _{\begin{array}{c} n \rightarrow \infty \\ r \rightarrow \infty \end{array}}\left\| F_r-F_n\right\| =0. \end{aligned}$$

Consequently, $\left\{ F_r\right\} _{r=0}^{\infty }$ in *B* is a Cauchy sequence. It follows that the series solution of Eq. (12) is convergent. $\square$

### Theorem 4.2

*If* $\sum _{k=0}^n \vartheta _k(x_{1}, \eta )$ *represents the approximate series solution of Eq*. (4), *then maximal absolute error can be determined by*

17 $$\begin{aligned} \left\| \vartheta (x_{1}, \eta )-\sum _{k=0}^n \vartheta _k(x_{1}, \eta )\right\| \le \frac{\delta ^{n+1}}{1-\delta }\left\| \vartheta _0(x_{1}, \eta )\right\| , \end{aligned}$$

*in which* $\delta$ *is a digit which means* $\dfrac{\left\| \vartheta _{i+1}\right\| }{\left\| \vartheta _i\right\| } \le \delta$.

### Proof

Using Eq. (15) from Theorem (4.1), we obtain

18 $$\begin{aligned} \left\| F_r-F_n\right\| \le \beta \left\| \vartheta _0(x_{1}, \eta )\right\| , \text{ in } \text{ which }\ \beta =\frac{\left( 1-\delta ^{r-n}\right) }{(1-\delta )} \delta ^{n+1} . \end{aligned}$$

Here, $\left\{ F_r\right\} _{r=0}^{\infty } \rightarrow \vartheta (x_{1}, \eta )$ as $r \rightarrow \infty$ and from Eq. (13), we get $F_n=\sum _{k=0}^n \vartheta _k(x_{1}, \eta )$,

19 $$\begin{aligned} \left\| \vartheta (x_{1}, \eta )-\sum _{k=0}^n \vartheta _k(x_{1}, \eta )\right\| \le \beta \left\| \vartheta _0(x_{1}, \eta )\right\| , \end{aligned}$$

Now, $$(1-\delta ^{r-n})<1$$, since $$0<\delta <1$$

20 $$\begin{aligned} \left\| \vartheta (x_{1}, \eta )-\sum _{k=0}^n \vartheta _k(x_{1}, \eta )\right\| \le \frac{\delta ^{n+1}}{1-\delta }\left\| \vartheta _0(x_{1}, \eta )\right\| . \end{aligned}$$

$$\square $$

Hence, the proof.

## Numerical applications

We provide some numerical tests for showing the precision and reliability of NIS. We can observe that, as compared to other approaches, this method is substantially easier to apply in obtaining the convergence series. We illustrate the physical nature of the resulting plot distribution with graphical structures. Furthermore, a visual depiction of the error distribution demonstrated the near correspondence between the NIS outcomes and the precise results. We can compute the absolute error estimates by evaluating the exact solutions with the NIS values.

### Example 1

Consider the one dimensional wave equation

21 $$\begin{aligned} \frac{\partial ^{2} \vartheta }{\partial \eta ^{2}}=\frac{\partial ^{2} \vartheta }{\partial x_{1}^{2}}-3 \vartheta , \end{aligned}$$

subjected to initial

22 $$\begin{aligned} \vartheta (x_{1},0)=0, \qquad \vartheta _{\eta } (x_{1},0)=2\cos (x_{1}) \end{aligned}$$

and boundary conditions

23 $$\begin{aligned} \vartheta (0,\eta )&=\sin (2 \eta ), \qquad \vartheta _{x_{1}} (\pi , \eta )=-\sin (2 \eta ). \end{aligned}$$

Apply $\mathbb {S}$T on Eq. (21), we get

$$\begin{aligned} \mathbb {S}\Big [\frac{\partial ^{2} \vartheta }{\partial \eta ^{2}}\Big ]=\mathbb {S}\Big [\frac{\partial ^{2} \vartheta }{\partial x_{1}^{2}}-3 \vartheta \Big ], \end{aligned}$$

Using the formula as defined in Eq. (3), it yields

$$\begin{aligned} \frac{Q(\theta )}{\theta ^{2}}-\frac{\vartheta (0)}{\theta ^{3}}-\frac{\vartheta '(0)}{\theta ^{2}}=\mathbb {S}\Big [\frac{\partial ^{2} \vartheta }{\partial x_{1}^{2}}-3 \vartheta \Big ]. \end{aligned}$$

Thus, $Q(\theta )$ reveals as

24 $$\begin{aligned} Q[\theta ]=\frac{\vartheta (0)}{\theta }+ \vartheta '(0)+\theta ^{2} \mathbb {S}\Big [\frac{\partial ^{2} \vartheta }{\partial x_{1}^{2}}-3 \vartheta \Big ]. \end{aligned}$$

On inverse $\mathbb {S}$T, we have

$$\begin{aligned} \vartheta (x_{1},\eta )=\vartheta (x_{1},0)+ \eta \vartheta _{\eta }(x_{1},0)+\mathbb {S}^{-1}\Big [\theta ^{2} \mathbb {S}\Big \{\frac{\partial ^{2} \vartheta }{\partial x_{1}^{2}}-3 \vartheta \Big \}\Big ]. \end{aligned}$$

Thus HPS yields such as

$$\begin{aligned} \sum _{i=0}^{\infty }p^{i}\vartheta _{i} (x_{1}, \eta )=2 \eta \cos (x_{1})+\mathbb {S}^{-1}\left[ \theta ^{2} \mathbb {S}\left\{ \sum _{i=0}^{\infty }p^{i}\frac{\partial ^{2}\vartheta _{i}}{\partial x_{1}^{2}}-3\sum _{i=0}^{\infty }p^{i} \vartheta \right\} \right] . \end{aligned}$$

By assessing comparable components of *p*, we arrive at

$$\begin{aligned} p^{0}&:\vartheta _{0} (x_{1}, \eta )=\vartheta (x_{1}, 0)=2 \eta \cos (x_{1}),\\ p^{1}&:\vartheta _{1} (x_{1}, \eta )=\mathbb {S}^{-1}\left[ \theta ^{2} \mathbb {S}\left\{ \frac{\partial ^{2}\vartheta _{0}}{\partial x_{1}^{2}}-3 \vartheta _{0}\right\} \right] = - \frac{(2 \eta )^{3}}{3!} \cos (x_{1}),\\ p^{2}&:\vartheta _{2} (x_{1}, \eta )=\mathbb {S}^{-1}\left[ \theta ^{2} \mathbb {S}\left\{ \frac{\partial ^{2}\vartheta _{1}}{\partial x_{1}^{2}}-3 \vartheta _{1}\right\} \right] = \frac{(2 \eta )^{5}}{5!} \cos (x_{1}),\\ p^{3}&:\vartheta _{3} (x_{1}, \eta )=\mathbb {S}^{-1}\Bigg [\theta ^{2} \mathbb {S}\bigg \{\frac{\partial ^{2}\vartheta _{2}}{\partial x_{1}^{2}}-3 \vartheta _{2}\bigg \}\Bigg ]=- \frac{(2 \eta )^{7}}{7!} \cos (x_{1}),\\ p^{4}&:\vartheta _{4} (x_{1}, \eta )=\mathbb {S}^{-1}\Bigg [\theta ^{2} \mathbb {S}\bigg \{\frac{\partial ^{2}\vartheta _{3}}{\partial x_{1}^{2}}-3 \vartheta _{3}\bigg \}\Bigg ]= \frac{(2 \eta )^{9}}{9!} \cos (x_{1}),\\&\vdots . \end{aligned}$$

Likewise, we can consider the approximation series in such a way that

25 $$\begin{aligned} \begin{aligned} \vartheta (x_{1},\eta )&=\vartheta _{0}(x_{1},\eta )+\vartheta _{1}(x_{1},\eta ) +\vartheta _{2}(x_{1},\eta )+\vartheta _{3}(x_{1},\eta )+\vartheta _{4}(x_{1},\eta )+\cdots ,\\&=\cos (x_{1})\Bigg (2\eta -\frac{(2 \eta )^{3}}{3!}+\frac{(2 \eta )^{5}}{5!}-\frac{(2 \eta )^{7}}{7!}+\frac{(2 \eta )^{9}}{9!} \Bigg )+\cdots . \end{aligned} \end{aligned}$$

**Table 1:** Error distribution of $\vartheta (x_{1}, \eta )$ along $x_{1}$-space at different values.

$x_{1}$	$\eta$	Analytical results	Precise results	Error distribution
0.25	0.2	0.377312	0.377312	00000
	0.4	0.695055	0.695055	00000
	0.6	0.903064	0.903064	00000
	0.8	0.968503	0.968499	4 $\times$10^-6^
	1	0.881078	0.88103	0.000048
0.50	0.2	0.341747	0.341747	00000
	0.4	0.629539	0.629539	00000
	0.6	0.817941	0.817941	00000
	0.8	0.877212	0.877208	4 $\times$10^-6^
	1	0.798627	0.797984	0.000043
0.75	0.2	0.284933	0.284933	00000
	0.4	0.524881	0.524881	00000
	0.6	0.681963	0.681963	00000
	0.8	0.73138	0.731377	3 $\times$10^-6^
	1	0.665359	0.665323	0.000036
1	0.2	0.210404	0.210404	00000
	0.4	0.387589	0.387589	00000
	0.6	0.503583	0.503583	00000
	0.8	0.540074	0.540072	2 $\times$10^-6^
	1	0.491323	0.491295	0.000028

**Figure 1:**
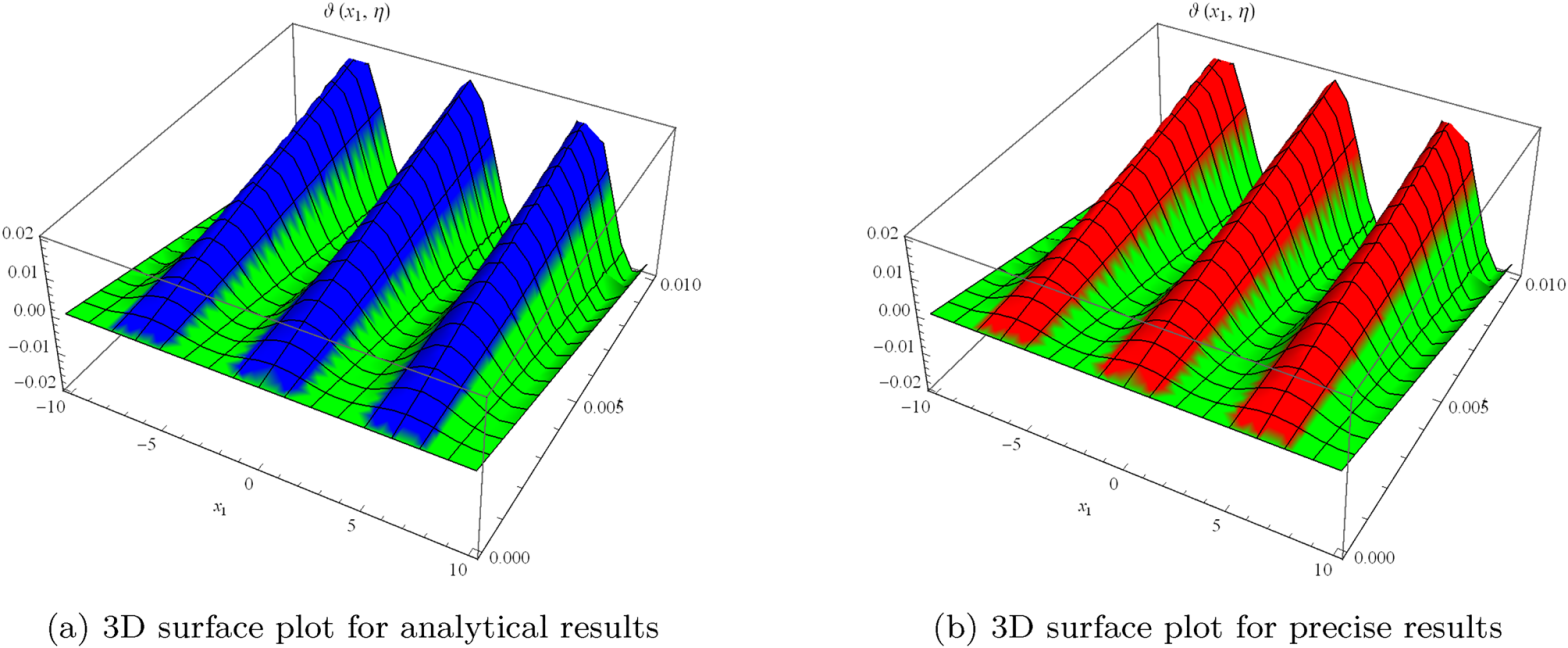
Surface results for one-dimensional problem.

**Figure 2:**
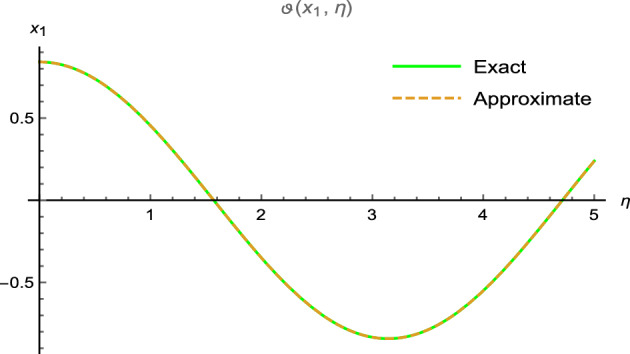
Error between analytical and precise results.

which can approaches to

26 $$\begin{aligned} \vartheta (x_{1},\eta )=\cos (x_{1})\sin (2\eta ). \end{aligned}$$

Figure 1 shows periodic soliton waves in two diagrams: Fig. 1a 3D surface plot for analytical results of $\vartheta (x_{1},\eta )$ and Fig. 1b shows 3D surface plot for precise results of $\vartheta (x_{1}, \eta )$ for one-dimensional wave equation at $-10\le x_{1} \le 10$ and $0\le \eta \le 0.01$. The effective agreement among analytical and the precise results at $0\le x_{1} \le 5$ along $\eta =0.1$ is shown in Fig. 2, which further validates the strong agreement of NIS for example (5.1). We can precisely propagate any surface to reflect the pertinent natural physical processes, according to this technique. The error distribution among analytical and precise results for $\vartheta (x_{1}, \eta )$ along $x_{1}$-space at different values is shown in Table 1. This contraction demonstrates the effectiveness of proposed technique in finding the closed-form results for the wave problems.

### Example 2

Consider the two-dimensional wave equation

27 $$\begin{aligned} \frac{\partial ^{2} \vartheta }{\partial \eta ^{2}}=2\Bigg (\frac{\partial ^{2} \vartheta }{\partial x_{1}^{2}}+\frac{\partial ^{2} \vartheta }{\partial y_{1}^{2}}\Bigg )+6 \eta +2 x_{1}+4 y_{1}, \end{aligned}$$

subjected to initial

28 $$\begin{aligned} \vartheta (x_{1},y_{1},0)=0, \qquad \vartheta _{\eta } (x_{1},y_{1},0)=2\sin (x_{1})\sin (y_{1}) \end{aligned}$$

and boundary conditions

29 $$\begin{aligned} \begin{aligned} \vartheta (0,y_{1},\eta )&=\eta ^{3}+2\eta ^{2}y_{1}, \qquad \vartheta _{x_{1}} (\pi ,y_{1}, \eta )=\eta ^{3}+\pi \eta ^{2}+2\eta ^{2}y_{1},\\ \vartheta (x_{1},0,\eta )&=\eta ^{3}+\eta ^{2}x_{1}, \qquad \vartheta _{x_{1}} (x_{1}, \pi , \eta )=\eta ^{3}+2\pi \eta ^{2}+\eta ^{2}x_{1}. \end{aligned} \end{aligned}$$

Apply $\mathbb {S}$T on Eq. (27), we get

$$\begin{aligned} \mathbb {S}\Big [\frac{\partial ^{2} \vartheta }{\partial \eta ^{2}}\Big ]=\mathbb {S}\Big [2\Big (\frac{\partial ^{2} \vartheta }{\partial x_{1}^{2}}+\frac{\partial ^{2} \vartheta }{\partial y_{1}^{2}}\Big )+6 \eta +2 x_{1}+4 y_{1}\Big ], \end{aligned}$$

Using the formula as defined in Eq. (3), it yields

$$\begin{aligned} \frac{Q(\theta )}{\theta ^{2}}-\frac{\vartheta (0)}{\theta ^{3}}-\frac{\vartheta '(0)}{\theta ^{2}}&=\mathbb {S}\Big [2\Big (\frac{\partial ^{2} \vartheta }{\partial x_{1}^{2}}+\frac{\partial ^{2} \vartheta }{\partial y_{1}^{2}}\Big )+6 \eta +2 x_{1}+4 y_{1}\Big ],\\ \frac{Q(\theta )}{\theta ^{2}}-\frac{\vartheta (0)}{\theta ^{3}}-\frac{\vartheta '(0)}{\theta ^{2}}&=\mathbb {S}\Big [2\Big (\frac{\partial ^{2} \vartheta }{\partial x_{1}^{2}}+\frac{\partial ^{2} \vartheta }{\partial y_{1}^{2}}\Big )\Big ]+6 \mathbb {S}\Big [ \eta \Big ]+2 x_{1} \mathbb {S}\Big [1\Big ]+4 y_{1} \mathbb {S}\Big [1\Big ], \end{aligned}$$

Thus, $Q(\theta )$ reveals as

30 $$\begin{aligned} Q[\theta ]=6 \theta ^{2}+2 x_{1} \theta +4 y_{1} \theta +\frac{\vartheta (0)}{\theta }+ \vartheta '(0)+\theta ^{2} \mathbb {S}\Big [2\Big (\frac{\partial ^{2} \vartheta }{\partial x_{1}^{2}}+\frac{\partial ^{2} \vartheta }{\partial y_{1}^{2}}\Big ]. \end{aligned}$$

On inverse $\mathbb {S}$T, we have

$$\begin{aligned} \vartheta (x_{1},y_{1},\eta )=\eta ^{3}+ x_{1} \eta ^{2}+2 y_{1} \eta ^{2}+\vartheta (x_{1},0)+ \eta \vartheta _{\eta }(x_{1},0)+\mathbb {S}^{-1}\Big [\theta ^{2} \mathbb {S}\Big \{2\Big (\frac{\partial ^{2} \vartheta }{\partial x_{1}^{2}}+\frac{\partial ^{2} \vartheta }{\partial y_{1}^{2}}\Big \}\Big ]. \end{aligned}$$

Thus HPS yields such as

$$\begin{aligned} \sum _{i=0}^{\infty }p^{i}\vartheta _{i} (x_{1},y_{1},\eta )=\eta ^{3}+ x_{1} \eta ^{2}+2 y_{1} \eta ^{2}+ 2\eta \sin (x_{1})\sin (y_{1})+\mathbb {S}^{-1}\Big [\theta ^{2} \mathbb {S}\Big \{2\Big (\sum _{i=0}^{\infty }p^{i}\frac{\partial ^{2}\vartheta _{i}}{\partial x_{1}^{2}}+\sum _{i=0}^{\infty }p^{i}\frac{\partial ^{2}\vartheta _{i}}{\partial y_{1}^{2}}\Big )\Big \} \Big ]. \end{aligned}$$

By assessing comparable components of *p*, we arrive at

$$\begin{aligned} p^{0}&:\vartheta _{0} (x_{1},y_{1},\eta )=\vartheta (x_{1}, 0)=\eta ^{3}+ x_{1} \eta ^{2}+2 y_{1} \eta ^{2}+ 2\eta \sin (x_{1})\sin (y_{1}),\\ p^{1}&:\vartheta _{1} (x_{1},y_{1},\eta )=\mathbb {S}^{-1}\Bigg [\theta ^{2} \mathbb {S}\bigg \{\frac{\partial ^{2}\vartheta _{0}}{\partial x_{1}^{2}}+\frac{\partial ^{2}\vartheta _{0}}{\partial y_{1}^{2}}\bigg \}\Bigg ]= - \frac{(2 \eta )^{3}}{3!} \sin (x_{1})\sin (y_{1}),\\ p^{2}&:\vartheta _{2} (x_{1},y_{1},\eta )=\mathbb {S}^{-1}\Bigg [\theta ^{2} \mathbb {S}\bigg \{\frac{\partial ^{2}\vartheta _{1}}{\partial x_{1}^{2}}+\frac{\partial ^{2}\vartheta _{1}}{\partial y_{1}^{2}}\bigg \}\Bigg ]= \frac{(2 \eta )^{5}}{5!} \sin (x_{1})\sin (y_{1}),\\ p^{3}&:\vartheta _{3} (x_{1},y_{1},\eta )=\mathbb {S}^{-1}\Bigg [\theta ^{2} \mathbb {S}\bigg \{\frac{\partial ^{2}\vartheta _{2}}{\partial x_{1}^{2}}+\frac{\partial ^{2}\vartheta _{2}}{\partial y_{1}^{2}}\bigg \}\Bigg ]=- \frac{(2 \eta )^{7}}{7!} \sin (x_{1})\sin (y_{1}),\\ p^{4}&:\vartheta _{4} (x_{1},y_{1},\eta )=\mathbb {S}^{-1}\Bigg [\theta ^{2} \mathbb {S}\bigg \{\frac{\partial ^{2}\vartheta _{3}}{\partial x_{1}^{2}}+ \frac{\partial ^{2}\vartheta _{3}}{\partial y_{1}^{2}}\bigg \}\Bigg ]= \frac{(2 \eta )^{9}}{9!} \sin (x_{1})\sin (y_{1}),\\&\vdots . \end{aligned}$$

Likewise, we can consider the approximation series in such a way that

31 $$\begin{aligned} \begin{aligned} \vartheta (x_{1},y_{1},\eta )&=\vartheta _{0}(x_{1},y_{1},\eta )+\vartheta _{1}(x_{1},y_{1},\eta )+\vartheta _{2}(x_{1},y_{1},\eta )+\vartheta _{3}(x_{1},y_{1},\eta )+\vartheta _{4}(x_{1},y_{1},\eta )+\cdots ,\\&=\eta ^{3}+ x_{1} \eta ^{2}+2 y_{1} \eta ^{2}+ \sin (x_{1})\sin (y_{1})\Bigg (2 \eta -\frac{(2 \eta )^{3}}{3!}+\frac{(2 \eta )^{5}}{5!}-\frac{(2 \eta )^{7}}{7!}+\frac{(2 \eta )^{9}}{9!} \Bigg )+\cdots . \end{aligned} \end{aligned}$$

**Table 2:** Error distribution of $\vartheta (x_{1}, y_{1}, \eta )$ along $x_{1}$-space and $y_{1}=0.5$ at different values.

$x_{1}$	$\eta$	Analytical results	Precise results	Error distribution
0.50	1	0.964469	0.964469	000000
	1.25	1.07034	1.07034	000000
	1.50	1.15241	1.15241	000000
	1.75	1.20946	1.20946	000000
	2	1.24183	1.24183	000000
1	1	3.36685	3.66683	000002
	1.25	3.66372	3.6637	000002
	1.50	3.93487	3.93481	000002
	1.75	4.17898	4.17896	000002
	2	4.39642	4.3964	000002
1.5	1	7.93362	7.93193	0.00169
	1.25	8.50361	8.50171	0.00190
	1.50	9.06949	9.06749	0.00200
	1.75	9.63105	9.62907	0.00198
	2	10.1883	10.1865	0.00180
2	1	15.733	15.6947	0.03830
	1.25	16.6989	16.6557	0.04320
	1.50	17.6835	17.6381	0.04540
	1.75	18.6878	18.643	0.04480
	2	19.7115	19.6701	0.04140

**Figure 3:**
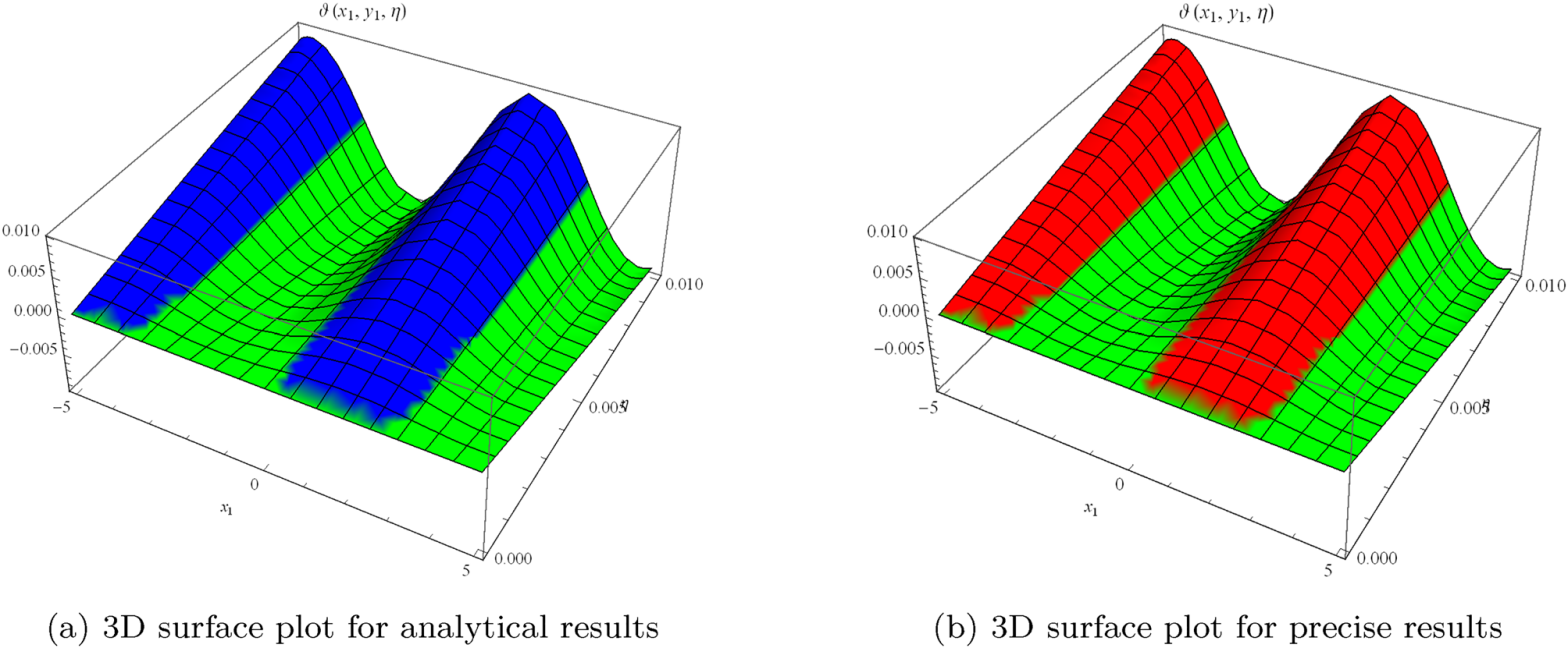
Surface results for two-dimensional problem.

**Figure 4:**
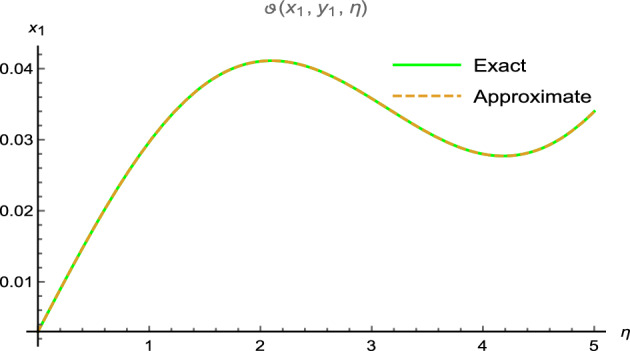
Error between analytical and precise results.

which can approaches to

32 $$\begin{aligned} \vartheta (x_{1},y_{1},\eta )=\eta ^{3}+ x_{1} \eta ^{2}+2 y_{1} \eta ^{2}+ \sin (x_{1})\sin (y_{1})\sin (2 \eta ). \end{aligned}$$

Figure 3 shows periodic soliton waves in two diagrams: Fig. 3a: 3D surface plot for analytical results and Fig. 3b: 3D surface plot for precise results of $\vartheta (x_{1},y_{1},\eta )$ for two-dimensional wave equation at $-5\le x_{1} \le 5$, $0\le \eta \le 0.01$ along $y_{1}=0.5$. The effective agreement among analytical and the precise results at $0\le x_{1} \le 5$, $y_{1}=0.1$ along $\eta =0.1$ is shown in Fig. 4, which further validates the strong agreement of NIS for example (5.2). We can precisely propagate any surface to reflect the pertinent natural physical processes, according to this technique. The error distribution among analytical and precise results for $\vartheta (x_{1},y_{1},\eta )$ along $x_{1}$-space at different values is shown in Table 2. This contraction demonstrates the effectiveness of proposed technique in finding the closed-form results for the wave problems.

### Example 3

Consider the three-dimensional wave equation

33 $$\begin{aligned} \frac{\partial ^{2}\vartheta }{\partial \eta ^{2}}=\frac{x_{1}^{2}}{18}\frac{\partial ^{2}\vartheta }{\partial x_{1}^{2}}+\frac{y_{1}^{2}}{18}\frac{\partial ^{2}\vartheta }{\partial y_{1}^{2}}+\frac{z_{1}^{2}}{18}\frac{\partial ^{2}\vartheta }{\partial z_{1}^{2}}-\vartheta , \end{aligned}$$

subjected to initial

34 $$\begin{aligned} \vartheta (x_{1},y_{1},z_{1},0)=0, \qquad \vartheta _{\eta }(x_{1},y_{1},z_{1},0)=x_{1}^{4}y_{1}^{4}z_{1}^{4}, \end{aligned}$$

and boundary conditions

35 $$\begin{aligned} \begin{aligned} \vartheta (0,y_{1},z_{1},\eta )=0, \qquad \vartheta (1,y_{1},z_{1},\eta )=y_{1}^{4}z_{1}^{4}\sinh (\eta ),\\ \vartheta (x_{1},0,z_{1},\eta )=0, \qquad \vartheta (x_{1},1,z_{1},\eta )=x_{1}^{4}z_{1}^{4}\sinh (\eta ),\\ \vartheta (x_{1},y_{1},0,\eta )=0, \qquad \vartheta (x_{1},y_{1},1,\eta )=x_{1}^{4}y_{1}^{4}\sinh (\eta ), \end{aligned} \end{aligned}$$

Apply $\mathbb {S}$T on Eq. (33), we get

$$\begin{aligned} \mathbb {S}\Big [\frac{\partial ^{2} \vartheta }{\partial \eta ^{2}}\Big ]=\mathbb {S}\Big [\frac{x_{1}^{2}}{18}\frac{\partial ^{2}\vartheta }{\partial x_{1}^{2}}+\frac{y_{1}^{2}}{18}\frac{\partial ^{2}\vartheta }{\partial y_{1}^{2}}+\frac{z_{1}^{2}}{18}\frac{\partial ^{2}\vartheta }{\partial z_{1}^{2}}-\vartheta \Big ]. \end{aligned}$$

Using the formula as defined in Eq. (3), it yields

$$\begin{aligned} \frac{Q(\theta )}{\theta ^{2}}-\frac{\vartheta (0)}{\theta ^{3}}-\frac{\vartheta '(0)}{\theta ^{2}}&=\mathbb {S}\Big [\frac{x_{1}^{2}}{18}\frac{\partial ^{2}\vartheta }{\partial x_{1}^{2}}+\frac{y_{1}^{2}}{18}\frac{\partial ^{2}\vartheta }{\partial y_{1}^{2}}+\frac{z_{1}^{2}}{18}\frac{\partial ^{2}\vartheta }{\partial z_{1}^{2}}-\vartheta \Big ] \end{aligned}$$

Thus, $Q(\theta )$ reveals as

$$\begin{aligned} Q[\theta ]=\frac{\vartheta (0)}{\theta }+ \vartheta '(0)+\theta ^{2} \mathbb {S}\Big [\frac{x_{1}^{2}}{18}\frac{\partial ^{2}\vartheta }{\partial x_{1}^{2}}+\frac{y_{1}^{2}}{18}\frac{\partial ^{2}\vartheta }{\partial y_{1}^{2}}+\frac{z_{1}^{2}}{18}\frac{\partial ^{2}\vartheta }{\partial z_{1}^{2}}-\vartheta \Big ]. \end{aligned}$$

On inverse $\mathbb {S}$T, we have

36 $$\begin{aligned} \vartheta (x_{1},y_{1},z_{1},\eta )=\vartheta (x_{1},0)+ \eta \vartheta _{\eta }(x_{1},0)+\mathbb {S}^{-1}\Big [\theta ^{2} \mathbb {S}\Big \{\frac{x_{1}^{2}}{18}\frac{\partial ^{2}\vartheta }{\partial x_{1}^{2}}+\frac{y_{1}^{2}}{18}\frac{\partial ^{2}\vartheta }{\partial y_{1}^{2}}+\frac{z_{1}^{2}}{18}\frac{\partial ^{2}\vartheta }{\partial z_{1}^{2}}-\vartheta \Big \}\Big ]. \end{aligned}$$

Thus HPS yields such as

$$\begin{aligned} \sum _{i=0}^{\infty }p^{i}\vartheta (x_{1},y_{1},z_{1},\eta )=\eta x_{1}^{4}y_{1}^{4}z_{1}^{4}+\mathbb {S}^{-1}\Big [\theta ^{2} \mathbb {S}\Big [\sum _{i=0}^{\infty }p^{i}\frac{x_{1}^{2}}{18}\frac{\partial ^{2}\vartheta _{i}}{\partial x_{1}^{2}}+\sum _{i=0}^{\infty }p^{i}\frac{y_{1}^{2}}{18}\frac{\partial ^{2}\vartheta _{i}}{\partial y_{1}^{2}}+\sum _{i=0}^{\infty }p^{i}\frac{z_{1}^{2}}{18}\frac{\partial ^{2}\vartheta _{i}}{\partial z_{1}^{2}}-\sum _{i=0}^{\infty }p^{i}\vartheta \Big ]. \end{aligned}$$

By assessing comparable components of *p*, we arrive at

$$\begin{aligned} p^{0}&:\vartheta _{0}(x_{1},y_{1},z_{1},\eta )=\vartheta (x_{1},y_{1},z_{1},0)=\eta x_{1}^{4}y_{1}^{4}z_{1}^{4},\\ p^{1}&:\vartheta _{1} (x_{1},y_{1},z_{1},\eta )=\mathbb {S}^{-1}\Bigg [\theta \mathbb {S}\bigg \{\frac{x_{1}^{2}}{18}\frac{\partial ^{2}\vartheta _{0}}{\partial x_{1}^{2}}+\frac{y_{1}^{2}}{18}\frac{\partial ^{2}\vartheta _{0}}{\partial y_{1}^{2}}+\frac{z_{1}^{2}}{18}\frac{\partial ^{2}\vartheta _{0}}{\partial z_{1}^{2}}-\vartheta _{0}\Big \}\bigg \}\Bigg ]=\frac{\eta ^{3}}{3!} x_{1}^{4}y_{1}^{4}z_{1}^{4},\\ p^{2}&:\vartheta _{2} (x_{1},y_{1},z_{1},\eta )=\mathbb {S}^{-1}\Bigg [\theta \mathbb {S}\bigg \{\frac{x_{1}^{2}}{18}\frac{\partial ^{2}\vartheta _{1}}{\partial x_{1}^{2}}+\frac{y_{1}^{2}}{18}\frac{\partial ^{2}\vartheta _{1}}{\partial y_{1}^{2}}+\frac{z_{1}^{2}}{18}\frac{\partial ^{2}\vartheta _{1}}{\partial z_{1}^{2}}-\vartheta _{1}\Big \}\bigg \}\Bigg ]=\frac{\eta ^{5}}{5!} x_{1}^{4}y_{1}^{4}z_{1}^{4},\\ p^{3}&:\vartheta _{3} (x_{1},y_{1},z_{1},\eta )=\mathbb {S}^{-1}\Bigg [2\theta \mathbb {S}\bigg \{\frac{x_{1}^{2}}{18}\frac{\partial ^{2}\vartheta _{2}}{\partial x_{1}^{2}}+\frac{y_{1}^{2}}{18}\frac{\partial ^{2}\vartheta _{2}}{\partial y_{1}^{2}}+\frac{z_{1}^{2}}{18}\frac{\partial ^{2}\vartheta _{2}}{\partial z_{1}^{2}}-\vartheta _{2}\Big \}\bigg \}\Bigg ]=\frac{\eta ^{7}}{7!} x_{1}^{4}y_{1}^{4}z_{1}^{4},\\ p^{4}&:\vartheta _{4} (x_{1},y_{1},z_{1},\eta )=\mathbb {S}^{-1}\Bigg [\theta \mathbb {S}\bigg \{\frac{x_{1}^{2}}{18}\frac{\partial ^{2}\vartheta _{3}}{\partial x_{1}^{2}}+\frac{y_{1}^{2}}{18}\frac{\partial ^{2}\vartheta _{3}}{\partial y_{1}^{2}}+\frac{z_{1}^{2}}{18}\frac{\partial ^{2}\vartheta _{3}}{\partial z_{1}^{2}}-\vartheta _{3}\Big \}\bigg \}\Bigg ]=\frac{\eta ^{9}}{9!} x_{1}^{4}y_{1}^{4}z_{1}^{4},\\&\vdots . \end{aligned}$$

Likewise, we can consider the approximation series in such a way that

37 $$\begin{aligned} \begin{aligned} \vartheta (x_{1},y_{1},z_{1},\eta )&=\vartheta _{0}(x_{1},y_{1},z_{1},\eta )+\vartheta _{1} (x_{1},y_{1},z_{1},\eta )+\vartheta _{2}(x_{1},y_{1},z_{1},\eta )+\vartheta _{3} (x_{1},y_{1},z_{1},\eta )+\vartheta _{4}(x_{1},y_{1},z_{1},\eta )+\cdots ,\\&=x_{1}^{4}y_{1}^{4}z_{1}^{4}\Big (\eta +\frac{\eta ^{3}}{3!}+\frac{\eta ^{5}}{5!} +\frac{\eta ^{7}}{7!}+\frac{\eta ^{9}}{9!}\Big )+\cdots . \end{aligned} \end{aligned}$$

**Table 3:** Error distribution of $\vartheta (x_{1}, y_{1}, z_{1}, \eta )$ along $x_{1}$ -space and $y_{1}=z_{1}=0.5$ at different values.

$x_{1}$	$\eta$	Analytical results	Precise results	Error distribution
0.25	1	0.0000179321	0.0000179321	00000
	1.25	0.0000244433	0.0000244433	00000
	1.50	0.0000324902	0.0000324902	00000
	1.75	0.0000425782	0.0000425783	1 $\times$10^-10^
	2	0.0000553407	0.0000553415	8 $\times$10^-10^
0.50	1	0.000286914	0.000286914	00000
	1.25	0.000391093	0.000391094	1 $\times$10^-10^
	1.50	0.000519843	0.000519844	1 $\times$10^-10^
	1.75	0.000681251	0.000681254	3 $\times$10^-10^
	2	0.000885451	0.00088564	13 $\times$10^-9^
0.75	1	0.0014525	0.0014525	00000
	1.25	0.00197991	0.00197991	00000
	1.50	0.00263171	0.00263171	00000
	1.75	0.00344883	0.00344885	2 $\times$10^-18^
	2	0.0044826	0.00448266	1 $\times$10^-7^
1	1	0.00459063	0.00459063	00000
	1.25	0.0062575	0.0062575	00000
	1.50	0.00831749	0.0083175	3 $\times$10^-7^
	1.75	0.0109	0.0109001	1 $\times$10^-7^
	2	0.0141672	0.0141674	2 $\times$10^-7^

**Figure 5:**
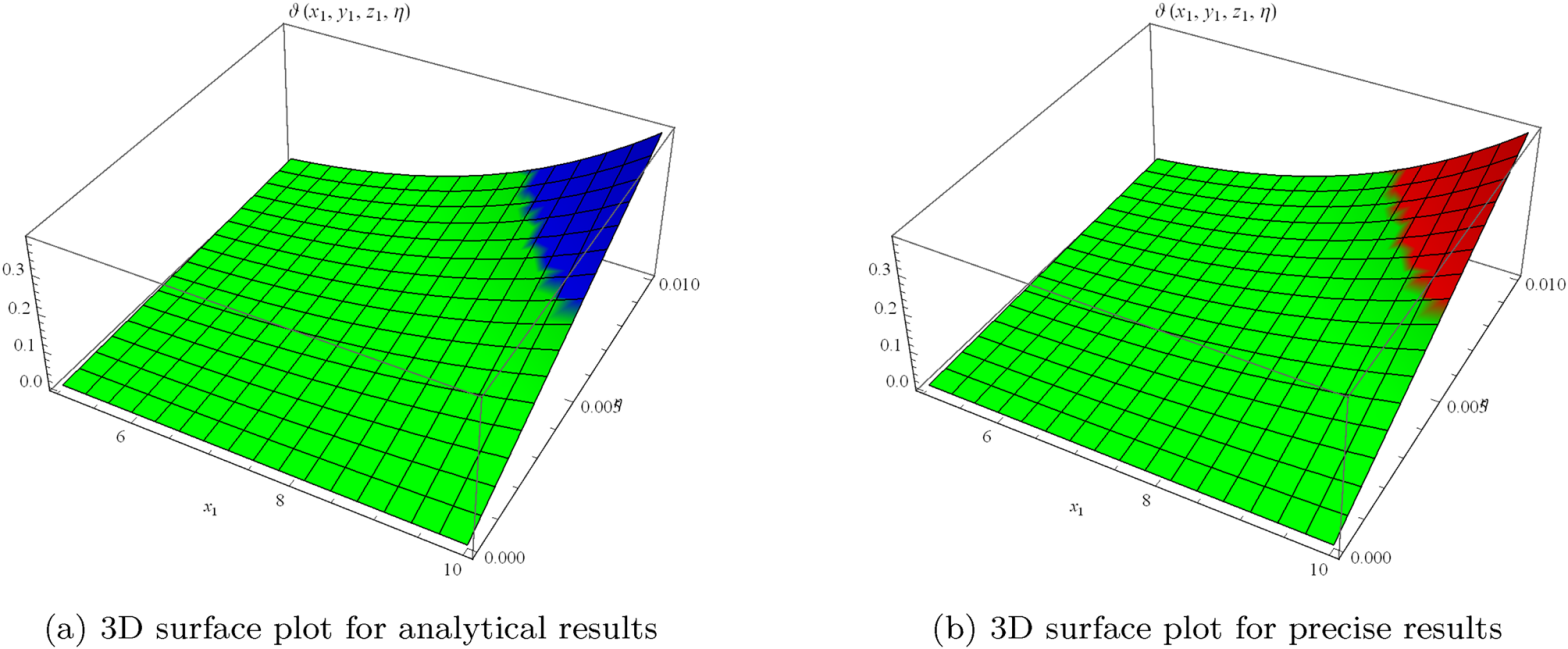
Surface results for three-dimensional problem.

**Figure 6:**
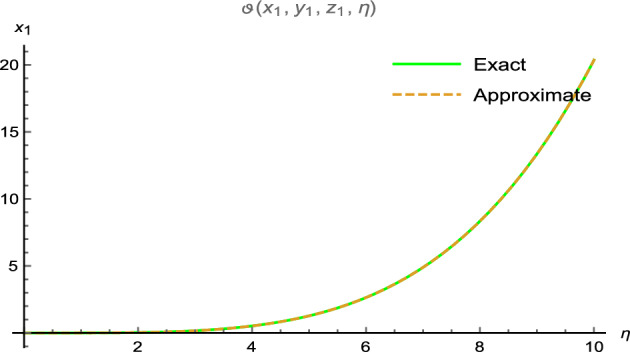
Error between analytical and precise results.

which can approaches to

38 $$\begin{aligned} \vartheta (x_{1},y_{1},z_{1}, \eta )=x_{1}^{4}y_{1}^{4}z_{1}^{4}\sinh (\eta ). \end{aligned}$$

Figure 5 shows two diagrams: Fig. 5a: 3D surface plot for analytical results and Fig. 5b: 3D surface plot for precise results of $\vartheta (x_{1},y_{1},z_{1},\eta )$ for two-dimensional wave equation at $5\le x_{1} \le 10$ and $0\le \eta \le 0.01$ with $y_{1}=0.5$ and $z_{1}=0.5$. The effective agreement among analytical and the precise results at $0\le x_{1} \le 10$, $y_{1}=0.5$, $z_{1}=0.5$ along $\eta =0.5$ is shown in Fig. 6, which further validates the strong agreement of NIS for example (5.3). We can precisely propagate any surface to reflect the pertinent natural physical processes, according to this technique. The error distribution among analytical and precise results for $\vartheta (x_{1},y_{1},z_{1},\eta )$ along $x_{1}$-space at different values is shown in Table 3. This contraction demonstrates the effectiveness of proposed technique in finding the closed-form results for the wave problems.

## Conclusion remarks and future work

In this article, we successfully applied the new iterative strategy for the approximate results of multi-dimensional wave problems. This technique uses the recurrence relation to produce the findings of the analysis. The findings obtained from numerical examples show that our technique is simple to implement and has a greater rate of convergence than existing approaches. The Sawi integral transform has the ability to control the global error, which makes it a suitable method for solving problems with rapidly changing solutions. The method is relatively easy to implement, especially for problems with periodic solutions. The 3D figures in the illustrated problems show the periodic soliton waves in the deep well. The physical behavior of the problems is depicted by the 3D graphical representations, and the visual inaccuracy between the exact outcomes and the produced results is represented by the 2D plot distribution. This method requires accurate initial guesses for the solution, which can be challenging in some cases. In terms of its effectiveness and efficiency, the Sawi integral transform is a relatively new method and has not been widely studied or compared to other numerical methods for solving PDEs. However, in the cases where it has been applied, it has shown promising results, with relatively high accuracy and efficiency compared to other methods. This composition of Sawi transform and the homotopy perturbation strategy gives the solution of multi-dimensional problems which is very useful in wave propagation. This novel iterative technique can also be used to solve other physical chemistry, engineering, and medical research challenges, such as calculating the growth rate of tumors, calculating the total quantity of infecting cells, calculating the amount of viral particles in blood during HIV-1 diseases, analyzing the impact of humidity on skew plate vibration, and calculating the amount of chemicals involved in chemical chain reactions in the future.

## Data Availability

This article includes all of the data from this study.
